# SILS Incisional Hernia Repair: Is It Feasible in Giant Hernias? A Report of Three Cases

**DOI:** 10.1155/2011/387040

**Published:** 2011-08-11

**Authors:** Umut Barbaros, Tugrul Demirel, Aziz Sumer, Ugur Deveci, Mustafa Tukenmez, Mehmet Ibrahim Cansunar, Murat Kalayci, Ahmet Dİnccag, Ridvan Seven, Selcuk Mercan

**Affiliations:** ^1^Department of General Surgery, Istanbul Faculty of Medicine, Istanbul University, 34452 Istanbul, Turkey; ^2^Department of General Surgery, Memorial Hizmet Hospital, 34452 Istanbul, Turkey; ^3^Department of General Surgery, Faculty of Medicine, Yuzuncuyıl University, 65080 Van, Turkey; ^4^Department of General Surgery, Faculty of Medicine, Maltepe University, 34845 Istanbul, Turkey; ^5^Department of General Surgery, Canakkale Military Hospital, 17000 Çanakkale, Turkey; ^6^Department of General Surgery, Faculty of Medicine, Yeditepe University, 34755 Istanbul, Turkey

## Abstract

*Aim*. Three incisional ventral abdominal wall hernias were repaired by placing a 20 × 30 cm composite mesh via single incision of 2 cm. *Methods*. All three cases had previous operations and presented with giant incisional defects clinically. The defects were repaired laparoscopically via single incision with the placement of a composite mesh of 20 × 30 cm. Nonabsorbable sutures were needed to hang and fix the mesh only in the first case. Double-crown technique was used in all of the cases to secure the mesh to the anterior abdominal wall. *Results*. The mean operation time was 120 minutes. The patients were mobilized and led for oral intake at the first postoperative day. No morbidity occurred. *Conclusion*. Abdominal incisional hernias can be repaired via single incision with a mesh application in experienced centers.

## 1. Introduction

The repair of ventral hernias has evolved from simple suture approximation to the use of prosthetic mesh and, recently, laparoscopic procedures. Laparoscopic ventral herniorrhaphy (LVH) was first described in 1993 by LeBlanc and Booth [1]. Since then, the minimally invasive operations have become more popular, due to improved patient outcomes reported in several studies [2–4]. Large series of laparoscopic ventral hernia repair have shown excellent results. 

As the laparoscopic approach had gained wide acceptance further minimally invasive techniques have been the main issues of argue. 

We here report a completely reproducible and safe technique for the repair of large incisional hernias purely performed through a single incision by “double-crown” mesh fixation technique.

## 2. Patients and Methods

All of the cases had previous operations and had giant incisional hernias. The first patient was a 63-year-old female patient who had an abdominal hysterectomy and bilateral salpingoophorectomy 3 years before for endometrial cancer via a median abdominal incision presented with a wide ventral incisional hernia. The second patient was a 65-year old male who had had a laparotomy via right subcostal incision for cholecystectomy. He referred us with a large incisional hernia, and the third patient was a 52-year-old woman who had a median laparotomy for ileus after an abdominal hysterectomy and bilateral salpingoophorectomy via open approach with a large hernia under the incisions.

## 3. How We Do It

The patients were entubated under general anesthesia. Gastric and bladder catheters were placed. All of the procedures were conducted via a flexible 3-channel port (SILS Port, Covidien, USA). The SILS port was placed in the middle between anterior superior iliac spine and costal margin on the left side in all patients. A 2 cm incision was made with the cutting mode of electrocautery device, and the peritoneal cavity was reached by open technique. A Sims-Maier sponge holder through the incision introduced the SILS port, and pneumoperitoneum of 12 mmHg was established. During the operation, articulating hand instruments of Covidien were used when needed besides the standard laparoscopic hand instruments. The peritoneal attachments of the hernia sac contents were carefully dissected free under direct vision as in the standard laparoscopic technique. We use 5 mm and 30° angled scope for SILS procedures. A special telescope with a flexible tip (Olympus Endo EYE) was used in the third case. After full mobilization and division of the adhesions, the defects were observed and measured by a scaled tape. The projections of the defect sides were signed on the abdominal skin, and four different signs were placed both to the abdominal wall and the mesh. The mesh was rolled and held by a grasper. The port was taken out, and the incision was cleaned by povidone-iodine. The composite mesh (HI-TEX COLEKIN IRPC 3D Composite Surgical Implant) was introduced into the abdomen, port was placed, and pneumoperitoneum was established again. The mesh was unrolled and lifted upwards to place the camera and second instrument underneath. The mesh was secured by 5 mm tacking device (Pro-tack Auto Suture; Tyco US, Norwolk, Conn, USA). In the first case, polypropylene sutures were used to hang and fix the mesh at the corners in order to ensure and secure the right positioning of mesh. In the latter two cases, no sutures were needed. Double-crown technique with at least 2 cm between the first and second crown was used in all of the cases to secure the mesh to the anterior abdominal wall. A suction drain was placed intra-abdominally. After the removal of the port, the defect was repaired with nonabsorbable thick suture materials, and the skin was closed with absorbable sutures. 

## 4. Results

The patients' demographics and data were shown in Table 1. There were no intraoperative complications. The mean operative time was 123.35 minutes. All of the procedures were completed via a three-channel single port with articulated and straight hand instruments. A 30 × 20 cm composite mesh was placed in all three cases. The patients were observed for few days before discharge for the drainage. All of them were discharged in less than five days (3, 4, and 4 days, resp.) without drains. One patient had a medical complication. He had a new atrial fibrillation that had to be treated by cardiologists in the first night after surgery. The patients were examined at the outpatient clinic in monthly scheduled appointments. None of them needed further imaging study, and no surgical complications were observed in short-term followup of 8 months so far.

## 5. Discussion

single incision laparoscopic surgery had entered the surgical era in the beginning of 1990's. M. A. Pelosi and M. A. Pelosi III had reported first single-port laparoscopic appendectomy in 1992 [5]. Only after a decade, single-port laparoscopic operations had been an important topic for various disciplines. Urologists, gynecologists, and widely general surgeons reported case reports or short series of single-port access laparoscopic procedures towards the end of the first decade of millennium [6–10].

Laparoscopic approach has been a standard intervention in anterior abdominal hernias both primary and incisional. As the experience cumulates, the papers on laparoscopic repair of large (>10 cm) defects are more frequently reported [11–16]. As in the standard use, one needs proper place to insert trocars, enough working space to liberate the adhesions, and availability for overlapping the mesh at least 4 cm over the defect. 

We routinely have contrast enhanced CT scans of the abdominal cavity in every patient preoperatively. The defects are precisely measured and mapped before the operation in order to predict the lateral sides of the defect. The site of insertion for the trocars is decided after CT examination. 

Single incision and/or single trocar laparoscopic abdominal wall hernia repairs were reported as case reports before [17, 18]. Mac Donald et al. reported a female patient who had a 5 × 6 cm ventral defect in the upper umbilical region presenting with irreducible hernia. They placed a multichannel port in the left flank and placed a 15 × 15 cm composite mesh. They used two transfacial sutures to fix the mesh besides multiple tacks. Bucher et al. reported a short series of 11 cases of ventral hernia (both primary and incisional). They introduced a standard laparoscopic port and used a working channel endoscope. They used only single straight laparoscopic instruments in every time. Therefore, they hung and fixed the meshes on four quadrants with transfacial fixing sutures. Only afterwards multiple tacks were used to fully fix the meshes. 

The defects in both reports were not large (>10 cm) or giant (15 cm). The authors used transfascial sutures in order to place the mesh properly and to fix it. We also thought that proper placement of the mesh with this much large dimensions inside the abdominal cavity without using the triangulation of instruments to aid would be very hard for the surgeon. So in the first case, we attached the polypropylene sutures in the four corners of the mesh. However, we easily placed the camera and instruments under the mesh. Positioning and fixing to the abdominal wall was not challenging. But hence we have had the sutures; we decided to use them to fix the mesh to the abdominal wall.

In the latter two cases, no sutures were used. The vital point for the repair of the giant hernias via single incision is the precise detection of the defect on the anterior abdominal wall. Abdominal contrast-enhanced CT scans are helpful in deciding the port placement. 

Defects such as subcostal incisions which are on the lateral parts of the anterior abdominal wall leave the operator more free space for the port placement. However, one may need to further take down the colon to comfortably lay the mesh over the lateral border of the defect. 

All three patients had narcotic analgesics once a day at the postoperative first and second days. 

Closure of single 2 cm port site is easier than securing all tree port sites of 5 and 10 mm trocars. But the issue needs more randomized controlled studies done to state that port site hernias would be less than standard laparoscopic trocar site hernias. 

In conclusion, repair of giant incisional hernias can be done as safely as multiport laparoscopic approach in experienced centers.

## Figures and Tables

**Table 1:** The patients' demographics and data.

P	A	S	PP	I	DL	OT	H	C	N (Day)
1	63	F	Abdominal hysterectomy	Median	18 cm	120 min	3	—	1
2	65	M	Cholecystectomy	Subcostal	17 cm	115 min	4	AF	1
3	52	F	Abdominal hysterectomy and bowel resection	Median and pfannenstiel	15 cm	135 min	4	—	1

P: patients, A: age, S: sex, PP: previous procedure, I: type of incision, DL: defect length, OT: operation time, H: hospitalization, C: complications, N: narcotic use.
